# Effect of β-Glucan Supplementation on Acute Postprandial Changes in Fatty Acid Profile of Lymph and Serum in Pigs

**DOI:** 10.3390/ijms150813881

**Published:** 2014-08-11

**Authors:** Helle Nygaard Lærke, Lasse Sommer Mikkelsen, Henry Jørgensen, Søren Krogh Jensen

**Affiliations:** 1 Department of Animal Science, Aarhus University, Blichers Allé 20, 8830 Tjele, Denmark; E-Mails: lasse_uha@yahoo.dk (L.S.K.); henry.jorgensen@agrsci.dk (H.J.); sorenkrogh.jensen@agrsci.dk (S.K.J.); 2 Læssøesgade 47 1.th, 8000 Århus C, Denmark

**Keywords:** lipid, absorption, dietary fibre, yeast, barley

## Abstract

Triglycerides are absorbed by the lymphatic system and have various functions in the body. It has been shown that some types of β-glucans have a positive effect on the systemic concentrations of cholesterol and lipid, presumably through interference with the absorption of lipid and/or reabsorption of bile acids. In the current study we investigated the acute effects of ingesting 2 g of β-glucan concentrates derived from barley β-(1→3)(1→4)-d-glucan or yeast β-(1→3)(1→6)-d-glucan on fatty acid content and composition in lymph and serum of 10 female pigs (initial weight 34.7 ± 1.1 kg) fitted with a permanent catheter in the jejunal lymphatic trunk in a cross-over design. Lymph was collected continuously for 8 h followed by a spot sample taken 24 h after. A significant effect of time after feeding was observed for all fatty acids in serum and for 18:0, 18:2ω6 and 18:3ω3 in lymph, but a significant effect of β-glucan was only observed for 14:0 (*p* = 0.049) and 22:6ω3 (*p* = 0.048) in lymph and 18:0 (*p* = 0.019) in serum. While the concentration of dietary fatty acids increased postprandially in lymph, the concentration of arachidonic and docahexanoic acid tended to decrease. Furthermore, there was a drop in concentration of all fatty acid in serum 1 h after the meal.

## 1. Introduction

It is well known that elevated levels of cholesterol and low density lipoprotein (LDL)-cholesterol in serum in humans are linked to an increase in blood pressure and an increased risk of atherosclerosis and coronary heart disease [1]. There is strong evidence that some types of dietary fibres can reduce the concentration of cholesterol and LDL-cholesterol in blood or help in maintaining a reasonable level [2]. Many studies have specifically investigated the effect of β-glucans, and both animal and human studies have shown that β-(1→3)(1→4)-d-linked glucans from oat and barley reduce the concentration of cholesterol in serum [3,4,5], while the effect on serum triglycerides is controversial [6,7]. The effects of the cereal based β-glucans appear to relate to their solubility and viscosity elevating effects, which is believed to interfere with the digestion processes in the gastrointestinal tract by reducing the rate and extent of lipid absorption and interfering with the reabsorption of bile acids. In production animals, however, this interference with the digestive processes may have a positive effect on gut health but a negative impact in growth and productivity.

β-glucans from yeast are insoluble and non-viscous polysaccharides consisting of β-(1→3)(1→6)-linkages. Although some studies indicate that yeast derived β-glucans can also modulate serum lipids, the evidence is scarce [8].

Due to the practical and technical difficulties in obtaining lymph, very few studies have been conducted to study postprandial absorption of lipids and acute effects of dietary intervention on composition of lymph fluid. Using a previously described technique, where pectin was found to reduce the absorption of dietary cholesterol [9], the present study was undertaken to investigate how an acute dose of isolated β-glucan from barley or yeast affected the content composition of fatty acids in lymph and serum. Previous observations from our group using ^1^H NMR-based metabonomics indicate that the levels of mono- and poly-unsaturated lipids were reduced by the β-glucan enriched diets [10]. 

## 2. Results

In general, dietary intervention only had a significant effect on few fatty acids in both lymph and serum, and no interaction was observed between time and diet in neither lymph nor serum.

### 2.1. Fatty Acids in Lymph

**Figure 1:**
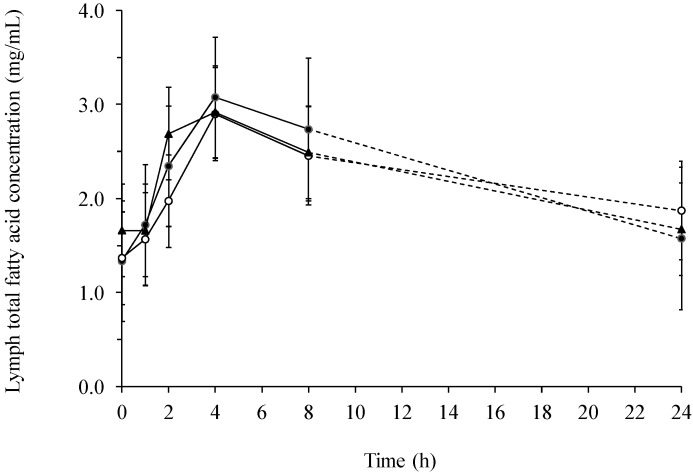
Total fatty acid concentration (mg/mL) in lymph of pigs fed a β-glucan free basal diet (●) or diets supplemented with either barley (○) or yeast (▲) β-glucan.

**Figure 2:**
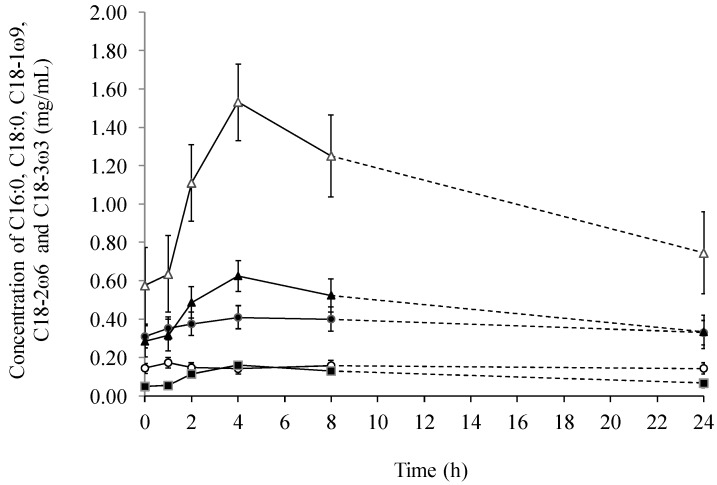
The average lymphatic concentration (mg/mL) of palmitic (16:0, ●), stearic (18:0, ○), oleic (18:1ω9, ▲), linoleic (18:2ω6, ∆) and linolenic (18:3ω3, ■) acid across diets over time.

There was a significant (*p* = 0.0008) change in the total fatty acid concentration with time after feeding (Figure 1), but with no effect of diet. The total fatty acid concentration raised 76%–131% from 0–4 h after ingestion, followed by a steady decrease from 4–24 h after ingestion approximating the concentration measured before feeding. The increase in total fatty acid content of lymph was mainly due to a big increase in the content of 18:1ω9 (*p* = 0.0001) and 18:2ω6 (*p* < 0.0001), but also 18:3ω3 increased significantly (*p* < 0.0001, Figure 2). In the first 4 h after feeding the concentration of 18:1ω9, 18:2ω6 and 18:3ω3 increased 120%, 166% and 231%, respectively.

**Figure 3:**
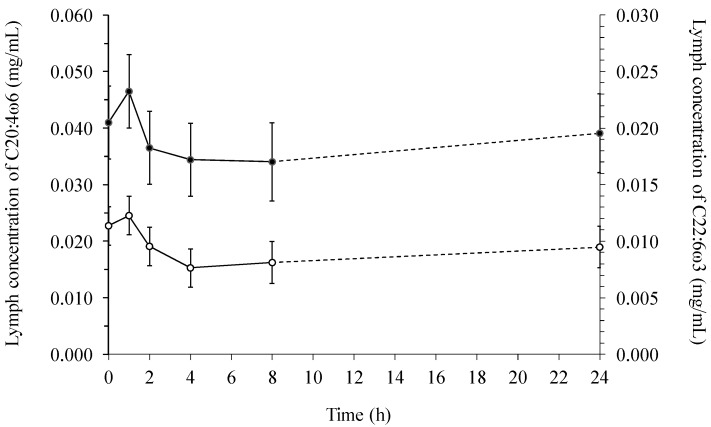
The average lymphatic concentration (mg/mL) of arachidonic (C20:4ω6, ● left) and docosahexaenoic (C22:6ω3, ○ right) acid across diets over time.

Arachidonic (C20:4ω6) and docosahexaenoic acid (C22:6ω3) appeared to drop 2–8 h postprandially after a modest initial increase (Figure 3), and was statistical independent of feeding time or diet in lymph.

Only a significant effect of diet was observed for myristic (14:0) acid (*p* = 0.049) and docosahexaenoic (22:6ω3) acid (*p* = 0.048). For 14:0, the concentration was significantly lower with the barley β-glucan supplemented diet (0.080 ± 0.031 mg/mL) compared to the basal diet (0.160 ± 0.038 mg/mL), when averaged over time. For 22:6ω3, the average concentration was significantly lower in the basal diet (0.080 ± 0.017 mg/mL) compared to the yeast β-glucan supplemented diet (0.120 ± 0.013 mg/mL).

### 2.2. Fatty Acid Contents in Serum

**Figure 4:**
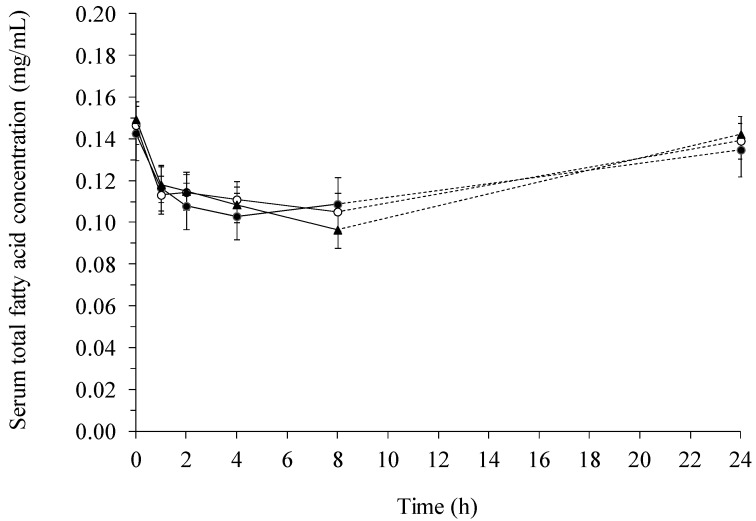
Total fatty acid concentration (mg/mL) in serum of pigs fed a β-glucan free basal diet (●) or diets supplemented with either barley (○) or yeast (▲) β-glucan.

The total fatty acid concentration in serum developed quite differently from the total fatty acid concentration in lymph. In serum, there was a significant reduction in the content of fatty acids after the meal (Figure 4). No significant interaction between diet and time was observed, and only a significant effect of diet was found for stearic (18:0) acid (*p* = 0.019), where the basal diet resulted in a significantly lower average concentration (0.15 ± 0.01 mg/mL) compared to the yeast diet (0.17 ± 0.01 mg/mL).

**Figure 5:**
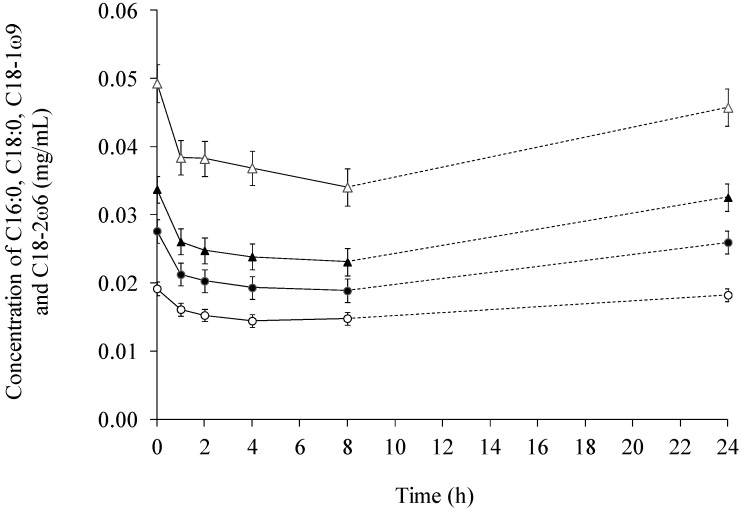
The concentration (mg/mL) of palmitic (16:0, ●), stearic (18:0, ○), oleic (18:1ω9, ▲), and linoleic (18:2ω6, ∆) in serum across diets over time.

**Figure 6:**
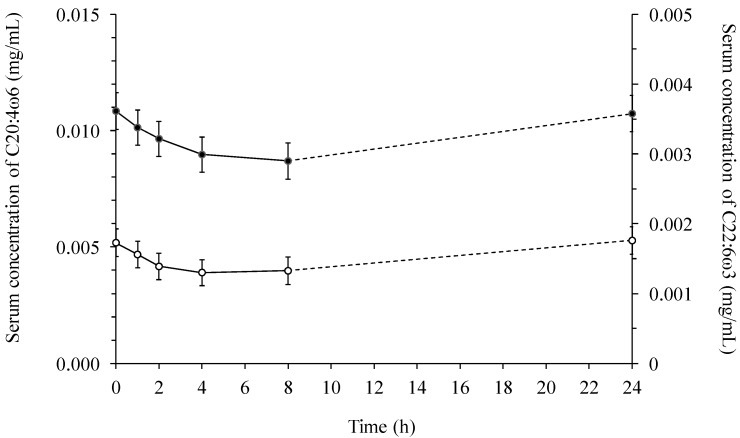
The concentration (mg/mL) of arachidonic (C20:4ω6, ● left) and docosahexaenoic (C22:6ω3, ○ right) acid in serum across diets over time.

The changes in the concentration of individual fatty acids in serum are depicted in Figure 5 and Figure 6. The effect of time was highly significant (*p* < 0.0005) for all fatty acids, except for C22:6ω3, where the effect was significant at a level of *p* = 0.036. A decrease in the concentration of the fatty acids was seen 1–4 h after feeding. Subsequently, the concentration in the basal diet steadily increased in the 4–24 or 8–24 h interval. At the 24 h sampling point, the initial fatty acid concentration was almost attained for all the diets.

In compliance with the changes observed for the concentration of the other fatty acids in serum, a convective shape for the concentrations of 20:4ω6 and 22:6ω3 over time was obtained (Figure 6). An approximate concentration was observed for the initial concentration (0.017 ± 0.002 mg/mL). The decrease from the initial concentration of 20:4ω6 and 22:6ω3 to the 4 h sampling point was calculated to be 20% and 25%, respectively.

When comparing lymph with serum it was found, that the total fatty acid concentration was several multiples greater in lymph than in serum (Figure 1 and Figure 4). In addition, the concentration of 18:2ω6 was the highest among all the fatty acids both in lymph (Figure 2) and serum (Figure 5), constituting 39%–52% of the total fatty acid content in lymph and 32.9%–34.3% in serum, depending on sampling time. Additionally, it was observed, that 22:6ω3 was in lowest concentration among the fatty acids, only comprising 0.26%–0.78% and 1.18%–1.35% of the total fatty acid concentrations in lymph (Figure 3) and serum (Figure 6), respectively.

## 3. Discussion

The current study demonstrated postprandial changes in the concentration and composition of fatty acids in lymph and serum, but very little effect of inclusion of β-glucan from barley or yeast in the diet was seen. This is in contrast to the study of Larsen *et al.* [10], where ^1^H NMR analysis suggested that β-glucans from barley reduced the absorption of both mono- and polyunsaturated fatty acids. A reason for the discrepancy between the obtained results could be difference in sensitivity of the analysis. Measurements of fatty acids in vegetable oils have shown high consistency between the results obtained by GC and ^1^H NMR [11] suggesting that ^1^H NMR is a valid method to detect fatty acids. However, because lymph not only contains fatty acids, signals from other molecules might interfere or disturb the signals obtained in ^1^H NMR. Another difference was the way the statistical evaluation of the data was performed as ^1^H NMR spectra were averaged with one spectrum for each combination of time and diet to reduce pig-to-pig variation, while in the current study postprandial profiles from each pig were treated separately.

There was an opposite course for the concentration of the individual fatty acids in serum and lymph, which may be explained by the natural correlation between the lymphatic and circulatory systems; lipids are absorbed to the lymphatic system, and eventually lymph is transferred to the circulatory system. Accordingly, a rise in the concentration of fatty acids in the lymphatic system would first be observed, and later a similar observation for the circulatory system. In the attempt to emulsify and digest fatty acids, bile acids are needed. Bile acids are synthesized from cholesterol, which also need fatty acids for synthesis. The drastic decrease of fatty acids in serum could possibly be explained by a higher bile acid synthesis in various cells, which lead to a higher uptake of fatty acids from the circulatory system.

The increase in the concentration of the dietary fatty acids (16:0, 18:0, 18:1ω9, 18:2ω6 and 18:3ω3) in lymph relates directly to the amount of the individual fatty acids in the diet, thus determining the amount available for absorption. The development in the systemic fatty acids (20:4ω6 and 22:3ω3) in both serum and lymph is not necessarily directly linked to the concentration of dietary ω6 and ω3 fatty acids, since these fatty acids although synthesized from ω6 and ω3 fatty acids has been through several regulatory steps during their synthesis.

In line with the present study, Casiraghi *et al.* [12] found that neither insoluble whole wheat bran nor barley rich in soluble β-glucans reduced the concentration of triglycerides in plasma in an acute study. In contrast, a meta-analysis showed that β-glucans derived from barley lowered the concentration of triglycerides in plasma [7]. A substantial difference between the meta-analysis and the present study is that the studies included in the meta-analysis, examined the long term effect of β-glucans on triglycerides opposed to the acute effect tested in the current study. Secondly, in the present study, the animals were normolipiedemic, and the total fat content of the diet rather low.

Ikeda *et al.* [13] found that certain dietary fibres (chitosan, guar gum and cellulose) were able to reduce absorption of cholesterol and triglycerides in lymph-cannulated rats, though the reduction was also dependent on the type of lipid source applied.

As previously mentioned, β-glucans may reduce absorption of bile acids and lipids by an increased viscosity of gut contents. This mechanism was studied in a recent *in vitro* experiment, which experiment demonstrated that both β-glucans of high viscosity and low viscosity from barley and oat reduced the intestinal absorption of specific fatty acids, primarily linoleic acid (18:2ω6) [14]. The effect was observed both in jejunum and ileum, when the environment mimicked both a high and low resistance of the unstirred water layer. However, as also demonstrated in that study, the reduction was particularly striking at higher fatty acid concentrations, which may explain the lack of effect in the current study. It is likely, that increasing the fat content in the diet and using hyperlipidemic and/or cholesterolaemic animals would have enhanced the response leading to significant differences between the dietary treatments.

## 4. Experimental Section

### 4.1. Animals and Surgery

A total of 10 female cross-bred Danish Landrace × Yorkshire pigs with an initial weight of 34.7 ± 1.1 kg was used for the study. After an overnight fast, the pigs were fed 200 mL cream 3 h prior to surgery. Under continuous infusion anaesthesia with Dipivan^®^ (propofol, Astra-Zenica A/S, Albertslund, Denmark) and Haldid^®^ (Fentanyl, Janssen-Cilag A/S, Birkerød, Denmark) the pigs were catheterized in the jejunal lymphatic trunk and posterior vena cave making a lymphatic shunt essentially as described previously [9,15] except that we used Tygon catheters (Cole-Parmer, Vernon Hills, IL, USA) with ID 1.016/OD 1.778 mm for the lymph catheter, ID 1.270/OD 2.286 mm for the vein catheter, and Tygon/silicone tubing (ID 1.6/OD 3.26 mm, Cole-Parmer, Vernon Hills, IL, USA) for external connection of the catheters, and Tygon cuffs. Post-surgical medication included intramuscular injection of analgesia (Finadyne and Temgesic, Schering-Plough A/S, Ballerup, Denmark), and prophylactic treatment with Streptocillin Vet^®^ (Boehringer Ingelheim Danmark A/S, Copenhagen, Denmark) for 3 days. The pigs were housed in individual smooth-walled 3 m^2^ pens without access to straw or other β-glucan containing rooting material.

The animal experiment was conducted according to protocols approved by the Danish Animal Experiments Inspectorate and complied with the guidelines of the Danish Ministry and Justice concerning animal experimentation and care of animals under study.

### 4.2. Feeding and Sampling

Twice daily the pigs were provided 500 g of a β-glucan free diet (basal) with the following composition in g/kg; heat-treated maize 664, toasted soy bean meal 160, sucrose 100, soy bean oil 30, l-lysine (40%) 12.0, dl-methionine 1.4, l-threonine 1.82, NaCl 3.8, CaCO_3_ 8.9, Ca_2_HPO_4_ 16.4, and vitamin/micromineral mixture VA Vit SL/US antiox 2.0 (Vestjyllands Andel, Ringkøbing, Denmark). In addition, the pigs were given 1 L/day of 1% Opti-Jern^®^ (Nutriscan, Odder, Denmark) in water along with free access to tap water.

Test meals consisted of 200 g of the basal diet containing 1% yeast β-glucan (Macroguard^®^, Orffa Scandinavia, Vejen, Denmark) or barley β-glucan (Glucagel^®^, GraceLinc Ltd., Christchurch, New Zealand) followed by 300 g of the basal diet without β-glucan, or when provided the β-glucan free test meal only 200 + 300 g basal diet. The test meals should be ingested within 1 h.

During sampling, the lymph and vein catheters were disconnected, the vein catheter was filled with saline and sealed, and lymph was collected for 1 h before the morning meal, then in hourly intervals until 8 h after the meal. Following the last sampling, the vein catheter was flushed with 2 mL 1000 IE heparin, the catheters were re-connected, and the pigs were fed 500 g of the β-glucan free diet in the afternoon. The following morning, lymph was again sampled for 1 h 23.5–24.5 h after the test meal. Using this procedure the test diets were given every second day in an incomplete cross-over design, and tests diets were provided in randomized order and collections of lymph continued as long as the lymph flowed freely from the catheter. Hence, 10 profiles were obtained from 8 pigs for the barley β-glucan and 9 profiles from 7 pigs for the yeast β-glucan. For 3 pigs, lymph was collected when feeding only the Control diet. The lymph was collected in 50 mL Greiner tubes attached to the side of the pig using catheter bags taped to the right flank of the pig. After measuring the volume per hour, lymph was stored at −80 °C until further analysis.

### 4.3. Chemical Analysis

Fat from lymph and serum was extracted with chloroform and methanol, according to Bligh and Dyer [16]. The same volume (0.5 mL) of lymph and serum was applied. To this volume, 1 mL of chloroform with C17 (Uvasol^®^, Merck, Darmstadt, Germany) (5 mg/mL as internal standard) and 2 mL of methanol was added. The addition of chloroform and methanol to lymph or serum resulted in a monophase, which was shaken for 1 min. Afterwards, one millilitre water was added and shaken for 1 min. again. Lastly, two millilitres chloroform was added to the mixture, which again was shaken for 1 min. followed by centrifugation at 1000× *g* for 10 min. Precisely 1 mL of the chloroform phase containing the fatty acids was drawn for the preparation of fatty acid methyl esters followed by gas chromatrography analysis, as previously described by Jensen [17]. The chloroform phase was evaporated under a stream of nitrogen and subsequently added 0.8 mL NaOH/methanol solution. Following this, the tube was filled with nitrogen, sealed and located in an oven for 15 min. at 100 °C. After cooling of the mixture, a 1 mL BF_3_ solution was added. The tube was then refilled with nitrogen and placed in an oven for 45 min at 100 °C. After cooling of the sample, 2 mL heptane and 4 mL of a saturated NaCl solution was added. This was shaken on a vortex mixer and then centrifuged at 3000 rpm for 10 min, 1 μL of the heptane phase was injected into the GC.

The fatty acid methyl esters was analyzed by gas chromatrography (HP 6890 series GC system, Agilent Technologies, Palo Alto, CA, USA) equipped with an automatic on-column injector (HP 7673, Hewlett Packard, Palo Alto, CA, USA) (Split ratio 4325:1); a capillary column of 30 m × 320 μm inner diameter; 0.25 μm film thickness (Omegawax; Supelco 4-293-415, Supelco, Bellefonte, PA, USA) and a flame ionization detector. All the fatty acids were quantified on the same GC system.

### 4.4. Statistical Analysis

Statistical analysis was performed as repeated measurements using the mixed procedure of SAS (SAS Institute, Inc., Cary, NC, USA). The effect of diet and time and the interaction between diet and time on lymph and serum concentrations and distribution of fatty acids were analyzed using the following normal Mixed model:

*Y_ijk_* = µ + α*_i_* + *d_j_*_(*i*)_ + τ*_k_* + (ατ)*_ik_* + ε*_ijk_* (1)

where α*_i_*; τ*_k_* and (ατ)*_ik_* are fixed effects of treatment *i*, time *k*, and their interaction, respectively, *dj*_(*i*)_ is the random effect associated with the *j^th^* subject in group *i*, ε*_ijk_* is random error associated with the *j^th^* subject in group *i* at time *k*, *d_j_*_(*i*)_ independent individually distributed N (0, σ_s_^2^)and ε*_ijk_* N (0, σ^2^). Variables meeting the criteria of normal distribution are reported as lsmeans ± SE. Only data from 6 of the 10 pigs were included in the statistical analysis for both lymph and serum.

## 5. Conclusions

In summary, the study has provided information on the acute temporal development of the total and individual fatty acid concentrations in serum and lymph. No significant dietary effect was found, except for 18:0 in serum, 14:0 and 22:6ω3 in lymph. Hence, when feeding a low-fat diet to normolipidemic pigs inclusion of barley or yeast β-glucan had a marginal effect on the acute postprandial concentrations of fatty acids in lymph and serum. Postprandial fluctuations in fatty acid concentrations and composition were more related to absorptive and metabolic changes caused by the fat in the diet.

In order to elaborate and expand the knowledge of the interactions between β-glucans and dietary fatty acids, more research is needed regarding the acute effects of β-glucans. These future studies should focus both on exploring and revealing the intricate interaction of these dietary components within the gastrointestinal tract concerning both the physico-chemical properties of β-glucans, and the amount of β-glucans in the diet. These studies should also relate their findings to the mechanisms for the absorption of fatty acids to the lymphatic system. Additionally, further investigation and optimization of the applicability of ^1^H NMR as an ubiquitous and reliable method to detect fatty acids in lymph are needed. 
